# Differential Effect of Hyperglycemia on the Odds of Cancer Among the Adult Population of the United States

**DOI:** 10.7759/cureus.63061

**Published:** 2024-06-24

**Authors:** Parinaz Ayat, Diana Sawass Najjar, Hussam Alkaissi, Harjinder Gill, Jennifer Otey, Marwa AlFaraj, Samy I. McFarlane

**Affiliations:** 1 Internal Medicine, State University of New York (SUNY) Downstate Health Science University, New York City, USA; 2 Internal Medicine, National Institutes of Health (NIH) National Institute of Diabetes and Digestive and Kidney Diseases (NIDDK), Bethesda, USA; 3 Medicine, State University of New York (SUNY) Downstate Health Science University, New York City, USA; 4 Integrative Medicine, State University of New York (SUNY) Downstate Health Science University, New York City, USA; 5 Internal Medicine, Endocrine Division, State University of New York (SUNY) Downstate Health Science University, New York City, USA

**Keywords:** obesity, insulin resistance, ketogenic diet, hyperglycemia, prediabetes, dysglycemia, cancer, diabetes

## Abstract

Objective

Accumulating evidence indicates a relationship between diabetes and cancer risk, with obesity, insulin resistance, and hyperglycemia being implicated as the major underlying pathogenetic mechanisms of increased cancer risk among people with diabetes. We aim to assess the differential effect of dysglycemia (prediabetes and diabetes) on the strength of association (odds) of cancer amongst the adult US diabetic population.

Material and methods

We analyzed data from the 1997-2013 National Health Interview Survey (NHIS) dataset, which applies a multistage area probability sampling design. We used descriptive statistics and logistic regression analyses to test the strengths of the association between diabetes, prediabetes, and cancer before and after adjusting for major risk factors for cancer, including age and body mass index (BMI).

Results

A total of 722,532 individuals were surveyed, with a mean age of 47.18 ±0.3 years (±SEM) and a BMI of 26.9 ±0.01 kg/m^2^. Between 1997 and 2013, BMI increased from 26.0 to 27.4 kg/m^2^, the diabetes rate increased from 4.1% to 7.6%, and associated cancer rates increased from 6.6% to 9.0%. Body mass index was 27.1 vs. 26.8 kg/m^2^, P < 0.01, for those with and without cancer, respectively. The unadjusted odds ratio for cancer was 1.92 (1.78-2.08) (95% CI) and 2.20 (2.13-2.27) for prediabetes and diabetes, respectively. After adjusting for age, BMI, race, and cigarette smoking, the odds ratio for cancer was 1.12 (1.03-1.22), P < 0.01, and 1.15 (1.11-1.18), P <0.01, for prediabetes and diabetes, respectively.

Conclusion

Among US adults, the increasing rate of diabetes over the years was associated with an increased rate of cancer. Diabetes and prediabetes have a graduated effect on cancer risk. While obesity is generally implicated as an underlying pathophysiologic link between diabetes and cancer, our study showed a modest difference in BMI between those with and without cancer. In addition, the effect of diabetes and prediabetes on the odds of cancer persisted after adjusting for BMI. These data collectively suggest that hyperglycemia is an attractive pathophysiologic mechanism that may play a role in increasing the odds of cancer among diabetic and prediabetic populations. Our study is consistent with the accumulating evidence implicating hyperglycemia in the pathogenesis of cancer, where glucose is used in PET scanning to detect cancer (the Warburg effect), and the ketogenic diet appears to be useful in cancer management, enhancing the effect of chemotherapeutic agents.

## Introduction

Diabetes mellitus is a serious health problem all around the world. Among all known cases of diabetes, about 90%-95% are classified as type 2 diabetes, which is characterized by insulin resistance and increased risk of cardiovascular disease, the major cause of death among the diabetic population [1-3]. Patients with diabetes are at increased risk of cancer compared to the non-diabetic population [4,5]. Although insulin has predominantly metabolic effects, it also has weak direct and indirect mitogenic properties. Hyperinsulinemia ensues from a combination of genetic and environmental factors and is characterized by high fasting plasma insulin levels and an exaggerated insulin response to increased plasma glucose concentrations [6]. 

While diabetes has been well-established as a risk factor for certain cancers, the data on prediabetes and the risk of cancer are scarce [7, 8]. Prediabetes is defined as glycated hemoglobin (HbA1c) ranging between 5.7% and 6.4%, fasting plasma glucose ranging between 100 and 125 mg/dL, or two-hour plasma glucose after a 75-g oral glucose tolerance test ranging between 140 and 199 mg/dL, and higher values are used to diagnose diabetes [9]. As such, compared to diabetes, prediabetes can be viewed as a milder state of cardiometabolic dysfunction resulting from insulin resistance. Several treatment options are available and approved for diabetes, yet there are no FDA-approved agents for prediabetes, and thus, lifestyle modification remains the mainstay of treatment [10].

In this study, we aim to assess the role and differential effect of hyperglycemia (prediabetes and diabetes states) on the odds of cancer among the adult population, with the hypothesis that hyperglycemia is the major pathogenetic mechanism of diabetes-associated cancer. This hypothesis is consistent with the accumulating data from experimental models and clinical and epidemiological studies. Furthermore, mechanistic evidence stems from the benefit of the ketogenic diet (essentially a low-carbohydrate diet) as an adjuvant therapy for several types of cancer, which in theory results in lower insulin levels and potentially reduces the insulin mitogenic effect. Proper clinical trials and mechanistic studies are needed to prove such causality [11].

Additionally, around 100 years ago, the German physiologist Otto Warburg described cancer's avidity for glucose, a principle named after him as the "Warburg effect," which serves as the basis for the diagnosis of cancer using 2-[18F] fluoro-2-deoxyglucose positron emission tomography (FDG-PET). Furthermore, some tumors upregulate targets downstream from insulin receptors to increase their uptake of glucose for growth and proliferation [12, 13].

Our study was presented as an abstract at the New York American College of Physicians (NYACP) annual meeting.

## Materials and methods

The National Health Interview Survey (NHIS) is a cross-sectional household interview survey conducted annually since 1957 and monitors the health of the civilian, non-institutionalized U.S. population. Data were collected continuously throughout the year, from January to December. The sample is distributed across all 50 states and the District of Columbia, approximately proportional to population size. The sample design follows a probability design that permits representative sampling of households. One of the major strengths of NHIS is its ability to analyze health measures based on many demographic and socioeconomic characteristics by obtaining information on major illnesses and chronic conditions and linking these data to sociodemographic characteristics, including health insurance status and access to care [14].

In this study, we analyzed 722,532 individual data from the 1997-2013 NHIS dataset, which applies a multistage area probability sampling design to test the degree of association between diabetes and cancer before and after adjusting for major risk factors for cancer, including age, smoking, and body mass index (BMI). We used descriptive statistics with analysis of variance to assess the differences among the groups for continuous variables and chi-squared analysis to determine the frequency of categorical data. We also developed a logistic regression model to assess the strength of the association (odds ratios) of cancer among prediabetic and diabetic populations compared to non-diabetic populations. In the NHIS survey, participants were asked about cancer in the following manner: “Have you ever been told by a doctor or other health professional that you had cancer or a malignancy of any kind? Answers were no or yes and were used in this analysis. For diabetes, participants were asked, “Have you ever been told by a doctor or other health professional that you have diabetes or sugar diabetes?" Answers were no, yes, or borderline.

## Results

A total of 722,532 individuals (Table 1) between 1997 and 2013 were surveyed and had the following characteristics: mean age = 47.18 ±0.3 years (±SEM), with BMI equal to 26.9 ±0.01 kg/m^2^. During this time interval, BMI increased from 26.0 to 27.4 kg/m^2^, and the diabetes rate increased from 4.1% to 7.6, with associated cancer rates increasing from 6.6% to 9.0%. Compared to males, female participants reported higher rates of cancer (8.4% vs. 6.7%, P < 0.01). Among male respondents, the rate of cancer reported was 5.9%, 12.5%, and 14.2% for non-diabetic, prediabetic, and diabetic populations, respectively, P < 0.01. For female participants, the rate of cancer reported was 7.8%, 12.7%, and 14.2% for non-diabetic, prediabetic, and diabetic populations, respectively, P < 0.01.

**Table 1:** Demographic data of the participants in the study

Total (n = 722,532)	Non-diabetic (n = 674,618)	Prediabetic (borderline) (n = 5,969)	Diabetic (n = 41,945)	P-value	
Age (years) means±SEM	45.8±0.026	57.66±0.213	60.87±0.070	<0.001	
BMI (kg/m^2^) mean±SEM	26.5±0.007	29.3±0.077	30.1±0.030	<0.001	
BMI category					
Normal (<25 kg/m^2^)	41.40%	22.30%	18.40%	<0.001	
Overweight (25-29.9 kg/m^2^)	36.50%	35.80%	34.40%	<0.001	
Obese (>30 kg/m^2^)	22.00%	41.90%	47.20%	<0.001	
Race	N (%)	N (%)	N (%)	P-value	Total
White	519,719 (93.7%)	4,497 (0.8%)	30,326 (5.5%)	<0.01	554,542(100%)
Black	103,034 (91.2%)	1,034 (0.9%)	8,940 (7.9%)	<0.001	113,008 (100%)
Asian	29,913 (94.4%)	286 (0.9%)	1,472 (4.6%)	<0.001	31671 (100%)
Hispanic	139,345 (94.4%)	790 (0.5%)	7,481 (5.1%)	<0.001	147,616 (100%)
Non-Hispanic, White	535,179 (93.1%)	5,179 (0.9%)	34,460 (6.0%)	<0.001	574,818 (100%)
Poverty					
At or above the poverty threshold	464,163 (93.7%)	4,056 (0.8%)	27,008 (5.5%)	<0.001	495,227 (100%)
Below poverty threshold	94,665 (91.8%)	935 (0.9%)	7,468 (7.2%)	<0.001	598,295 (100%)

Table 1 presents demographic data, which shows the population divided based on their mean age, BMI, BMI category, race, and poverty in three groups: non-diabetics, borderline diabetics, and diabetics. During this time period, BMI increased from 26.0 to 27.4 kg/m^2^, and the diabetes rate increased from 4.1% to 7.6, with associated cancer rates increasing from 6.6% to 9.0%. Body mass index was found to be 27.1 kg/m^2^ in people with cancer vs. 26.8 kg/m^2^ in people without cancer (P < 0.01). There was a graded effect of obesity on prediabetes and diabetes, where BMI was 26.5±0.007 kg/m^2^ for non-diabetics, 29.3±0.077 kg/m^2^ for prediabetics, and 30.1±0.030 kg/m^2^ for the diabetic population (P < 0.01) (Table 1).

In our logistic regression models, the odds ratio of cancer (with a 95% confidence interval) is categorized based on age, smoking status, BMI, poverty, and diabetes status (Table 2). Module 1 shows the cancer odds ratio in prediabetic and diabetic patients prior to adjustments. Module 2 shows the cancer odds ratio in the prediabetic and diabetic populations after adjustments for age, BMI, smoking, and race. The odds of cancer remained significantly higher in hyperglycemia, 12% higher in the borderline diabetic populations, and 15% higher in diabetic patients when compared to non-diabetic populations (Table 2).

**Table 2:** Odds ratio (OR) of cancer, module 1 (unadjusted) and module 2 (adjusted for age, BMI, smoking, and race)

Characteristics	OR	95% CI	P-value
Age			
18-44 years old	1		
45-64 years old	4.3	4.14-4.42	<0.01
>65 years old	12.24	11.86-12.63	<0.01
Smoking status			
Never smokes	1		
Former smoker	2.26	2.216-2.32	<0.01
Currently smoker	1.03	1.009-1.06	<0.01
BMI			
Normal (<25 kg/m^2^)	1		
Overweight (25-29.9 kg/m^2^)	1.07	1.039-1.092	<0.01
Obese (>30 kg/m^2^)	1.104	1.065-1.126	<0.01
Poverty			
At or above the poverty threshold	1		
Below poverty threshold	0.75	0.73-0.77	<0.01
Diabetic module			
Non-diabetic	1		
Prediabetic module 1	1.92	1.78-2.08	<0.01
Diabetic module 1	2.2	2.13-2.27	<0.01
Prediabetic module 2	1.12	1.03-1.22	<0.01
Diabetic module 2	1.15	1.11-1.18	<0.01

## Discussion

Our current study, conducted on a large sample of 722,532 individuals included in the 1997-2013 NHIS, demonstrates increased odds of cancer with a graded effect between prediabetes and diabetic individuals. This study provides further support for previous data suggesting that, among the US adult population, the increasing rate of diabetes over the years was associated with an increased rate of several types of cancer. The overarching hypothesized pathogenetic mechanisms linking diabetes and cancer include obesity as an underlying common risk factor for both diabetes and cancer, insulin resistance, hyperinsulinemia, and hyperglycemia, which are also proposed as potential underlying mechanisms for the link between diabetes and cancer [15-17]. While epidemiological studies indicate that hyperglycemia and cancer risk may be linked, further lines of evidence regarding the link between hyperglycemia and cancer are also provided from preclinical studies and, more recently, clinical studies that have shown that the ketogenic diet (which is essentially a high-fat and low-carbohydrate diet) can decrease tumor growth, increase survival time, and enhance clinical responses to standard therapeutic interventions for cancer, including chemotherapy and radiation therapy [18-21].

Our results are consistent with data from a large population-based cohort study that included 383,799 subjects without diabetes and 23,358 with diabetes included in a cancer registry from 2010-2013 [22]. The investigator reported that the overall cancer incidence was higher in subjects with diabetes than in those without diabetes, with incidence rate ratios (IRR) = 1.22, 95% CI, 1.15-1.29. The study included several types of cancer, such as liver, pancreas, colon, rectum, and bladder, in both sexes [22]. Interestingly, this study also found higher rates of cancer with a longer duration of diabetes and also among insulin users, pointing out that poor-controlled hyperglycemia requiring insulin use might be a culprit for increased cancer risk [22], a finding that is also suggested by our current study. It is also important to note that in this large population-based study where prevalent diabetes at baseline was positively associated with total cancer incidence, the risk was slightly higher in females than in males [22], a finding that was also demonstrated in our study where females had a higher overall cancer rate of 8.4%, compared to 6.7% for male participants.

Our study is also consistent with the accumulating evidence implicating hyperglycemia in the pathogenesis of cancer, where glucose is actually used in PET scanning to detect cancer (the Warburg effect), indicating that anaerobic metabolism of glucose is a fundamental property of all tumors, even in the presence of an adequate oxygen supply [23].

While the exact mechanism of the increased risk of cancer is not clearly established, evidence suggests that hyperglycemia may result in DNA damage caused by oxidative stress and impaired DNA repair mechanisms [24].

Hyperglycemia also plays an important role in apoptosis resistance, increased cancer cell proliferation, and cancer progression [25]. Furthermore, hyperglycemia upregulates glucose transporters (GLUT1 and GLUT3), thus further amplifying glucose uptake in certain cancers, a potential molecular mechanism contributing to the adjunct effect of glucose in tumor therapy [26].

Finally, our study is limited by being based on a questionnaire with recall bias and a lack of specific data regarding the type and stage of cancer and the exact relationship with diabetes, whether occurring before or after cancer diagnosis. Furthermore, specific glycemic data such as HbA1C and blood glucose were not available, and the distinction between diabetes and pre-diabetes (borderline diabetes) was based on participant recall. Nevertheless, given the very large number of participants (> 700,000) and the high-quality design and conduct of the study, we believe that these data provide further insights into the link between diabetes and cancer and could serve as a hypothesis-generating study for further research necessary to advance the field.

## Conclusions

Among adults in the US population surveyed from 1997-2013, there has been an increasing rate of obesity, associated with an increasing rate of diabetes and cancer. While age and obesity have been implicated as the major risk factors for cancer, our data, adjusted for age, obesity, and smoking, provide further evidence linking the risk of cancer to diabetes, specifically hyperglycemia, given the graded effect of dysglycemia (prediabetes and diabetes) on the odds of cancer among the adult population in the US. These findings are important, as our study is the first to demonstrate such a graded effect of dysglycemia on cancer and also the first study to evaluate specifically the odds of cancer in the prediabetic population. While it does not provide direct evidence, our results are also consistent with the emerging data on the utility of the ketogenic diet (effectively a low-carbohydrate diet) as adjuvant therapy for various cancers. However, further studies are needed to elucidate the underlying molecular mechanisms of the association between dysglycemia and cancer, which are currently not completely understood.
